# Characterization of Functional Ingredients Extracted with Ethanol Solvents from Ponkan (*Citrus reticulata*) By-Products Using the Microwave Vacuum Drying Method Combined with Ultrasound-Assisted Extraction

**DOI:** 10.3390/foods13132129

**Published:** 2024-07-03

**Authors:** Yu-Wei Chang, Yen-Ling Chen, Sung Hoon Park, Encarnacion Emilia S. Yap, Wen-Chieh Sung

**Affiliations:** 1 Department of Food Science, National Taiwan Ocean University, Keelung 20224, Taiwan; bweichang@mail.ntou.edu.tw (Y.-W.C.); kelly412293@gmail.com (Y.-L.C.); 2 Department of Food and Nutrition, College of Life Science, Gangneug-Wonju National University, Gangneung 25457, Republic of Korea; sungpark@gwnu.ac.kr; 3 Seafood PRIME Laboratories, Institute of Fish Processing Technology, College of Fisheries and Ocean Sciences, University of the Philippines, Visayas Miagao, Iloilo 5023, Philippines; esyap@up.edu.ph; 4 Center of Excellence for the Oceans, National Taiwan Ocean University, Keelung 20224, Taiwan

**Keywords:** microwave vacuum drying, phenols, flavonoids, pectin, dietary fiber, antioxidant activity

## Abstract

For this study, microwave vacuum drying (MVD) was combined with ultrasound-assisted extraction to compare the effects of different ethanol volumes on ponkan extract and to evaluate the total phenolic content (TPC), total flavonoid content (TFC), and total ascorbic acid content (TAAC). High-performance liquid chromatography with photodiode array detection (HPLC-PDA) was used to analyze the flavanone contents and antioxidant activity of ponkan (*Citrus reticulata*) peels. The experimental results showed that the TPC and TFC increase with ethanol volume. Ethanol extraction (75%) showed significant advantages by increasing the TPC to 17.48 mg GAE/g (DW) and the TFC to 2.96 mg QE/g (DW) of ponkan extract and also exhibited the highest antioxidant activity. The TAAC improved along with increased water content. Water extraction showed the highest content (13.07 mg VitC/100 g, DW). The hesperidin content analyzed by HPLC-PDA was 102.95–622.57 mg/100 g (DW), which was the highest among the flavanones. Then, the ethanol insoluble residue extracts were taken from the pectin with four different solvents, evaluating TPC, TFC, and antioxidant activity. The TPC, TFC, and antioxidant capacity of pectin are significantly lower than those of the peels. Combining MVD and 75% ethanol with ultrasound-assisted extraction in the pre-treatment process can effectively eliminate polyphenols, flavonoids, and other compounds, thus enabling the extraction of high-methoxyl pectin. The total dietary fiber (TDF) content of MVD ponkan by-products was 25.83%. Ponkan by-products have the potential for the future development of functional foods and supplements.

## 1. Introduction

Of the world’s nearly 80 million tons of *citrus* produced annually, the juice industry generates a significant volume of by-products, such as exocarp, mesocarp, carpel, and seeds, which collectively account for half of that weight each year [1]. These by-products are mainly used as animal feed or directly discarded as environmental waste without proper treatment [2]. Citrus peels contain a wide variety of bioactive compounds and are regarded as a promising source of functional ingredients [3], such as phenolic acids, flavanones, flavonoids, polymethoxylated flavones (PMFs), carotenoids, ascorbic acid, and fibers [4]. They have antioxidant, antimicrobial, anticancer, antimutagenic, and antiallergic properties [5]. Therefore, the *citrus* by-products of the juice industry are an important source of phenolic compounds, which have potential significance as ingredients in dietary supplements, raw materials for cosmetics, natural additives in food, or applications in food products [6].

*Citrus* peels have tremendous economic value due to their richness in flavonoids, polyphenols, carotenoids, pectin, dietary fibers, sugars, essential oils, ascorbic acid, and functional ingredients. Additionally, they contain high levels of sugars that are suitable for fermentation to produce bioethanol [1]. Therefore, research emphasis has been placed on reusing *citrus* by-products to enhance their value.

Microwave vacuum drying (MVD) is achieved by the combination of microwave and vacuum systems, using microwaves to directly heat the center of the object, and, through the vacuum decompression of the environment, significantly shorten the drying time, improve the production efficiency and reduce oxidation of the material [7], prevent the structure shrinkage of tissues [8], and avoid the deterioration in quality caused by prolonged heating [9].

Ultrasound-assisted extraction (UAE) utilizes sound waves with a frequency higher than 20 kHz to induce mechanical vibration in solids, liquids, and gases. It involves cycles of expansion and compression during medium propagation [10]. Ultrasonic waves can penetrate the cell wall, resulting in cell rupture within a shorter period of time. This process increases the extraction volume [11] and lowers the operating temperature for extracting heat-intolerant compounds [12,13].

The aim and novelty of this study were to dry ponkan by-products using MVD, extract the functional ingredients with varying ethanol concentrations combined with UAE, determine the phytochemical content in ponkan by-products, analyze the flavanone content with HPLC-PDA, assess antioxidant activity, investigate the pectin extraction residue to enhance ingredient yield, reduce extraction time, and promote environmental friendliness, and analyze the physicochemical properties and antioxidant activity.

## 2. Materials and Methods

### 2.1. Raw Materials and Chemicals

Ponkan (*Citrus reticulata*) pomace contains exocarp, mesocarp, carpel, seeds, a small amount of juice, and pulp. The pomace used in this study was provided by the Chia Meei Food Industrial Corp. (Taichung, DaLi, capital of Taichung Prefecture, Taiwan). The pomace from the juice extracted by the factory was stored in the factory’s refrigerator at −20 °C. This pomace was used as the raw material for the experiment and was transported to the laboratory using frozen logistics, during which it was also stored at −20 °C.

Acetic acid, amyloglucosidase, ascorbic acid, carbazole, 1,1-diphenyl-2-picrylhydrazyl (DPPH), galacturonic acid, gallic acid, potassium persulfate, protease, quercetin dehydrate 98%, termamyl, hesperidin (≥99%), neohesperidin (≥95%) and naringenin (≥95%) (for the HPLC analysis) were purchased from Sigma Aldrich (St. Louis, Missouri, USA). Boric acid, 2,2′-azino-bis (3-ethylbenz thiazoline-6-sulphonic acid), ethoxyethane, hydrochloric acid, Kjeldahl tablets, sodium acetate, sodium di-hydrogen phosphate 2-hydrate, di-sodium hydrogen phosphate anhydrous, and sulfuric acid were purchased from PanReac Applichem (Darmstadt, Germany). Trolox was purchased from Acros Organics B.V.B.A., Wakefield, MA, USA. Acetone was purchased from J.T. Baker (Bridgewater, NJ, USA). Naringin hydrate (≥98%) (for the HPLC analysis) was supplied by Alfa Aesar (Ward Hill, MA, USA). All the chemical reagents used were of analytical grade.

### 2.2. Ponkan By-Product Samples before Processing

Ponkan pomace was dried in an MVD oven (Microwave Vacuum Drying Oven, VP-09AV, LanTai Microwave Equipment Co., Ltd., Shanghai, China) at 0.85 w/g power for the vacuum, with the temperature set at 63–65 °C. The drying process continued until the water activity reached 0.3 ± 0.05, as measured by a water activity meter (Pawkit Pocket, Smartec Scientific Corp., Laurel, MD, USA). The moisture content in wet weight was maintained below 5 ± 1%. The microwave vacuum-dried pomace was then ground into powder, sieved through a 40-mesh sieve, and finally stored in desiccators. The ponkan pomace was also pre-chilled at −20 °C, freeze-dried for 48 h, ground into powder using a grinder, and then filtered through a 40-mesh sieve, and was finally stored in a refrigerator at −20 °C. The ponkan pomace was then cut into 1 cm^3^ cubes and dried at 50 °C for 48 h. Subsequently, the pomace cubes were ground into powder with a grinder and filtered through a 40-mesh sieve to produce hot-air-dried samples.

The yield was calculated as shown in the following equation [14]:

Yield (%) = W_dry_ × 100/W_wet_ (%).

W_dry_ is the dry weight (g) of the pomace; W_wet_ is the wet weight (g) of the pomace. All sample values were determined in triplicate.

### 2.3. Proximate Analysis

The experiment was carried out according to the method of AOAC [15], which involved oven-drying at 105 °C until a constant weight was achieved for moisture determination. The Kjeldahl nitrogen method and Soxhlet extraction method were used to analyze crude protein and crude fat content, respectively. Ash content was determined using an ashing furnace, while the acid–alkali cooking method was employed for crude fiber analysis. Carbohydrate content was calculated using the deduction method. With this method, the total weight of the sample is set at 100%. After deducting moisture, crude protein, crude fat, and ash content, the carbohydrate content is then calculated.

### 2.4. Flavonoid Extraction

Different concentrations of ethanol (25%, 50%, 75%, and 95% *v*/*v*) were used to extract the ponkan by-products, with water serving as the control group. A 1-gram sample dried using a vacuum microwave was taken and mixed with 20 mL of water or ethanol solutions of 25%, 50%, 75%, or 95% (*v*/*v*). The mixture was then subjected to extraction using an ultrasonic shaking device at 28 kHz for 30 min, followed by centrifugation at 8000× *g* for 10 min. The resulting solution was filtered to eliminate solids, and the extracts were stored in a refrigerator at −20 °C [16].

### 2.5. Analysis of Phytochemical Content

#### 2.5.1. Total Phenolic Contents (TPC)

First, 0.01 mL of the extract was mixed with 0.99 mL of deionized water. Subsequently, 0.5 mL of Folin–Ciocalteu phenol reagent was added to the mixture and allowed to react for 3 min. Following this, 1.5 mL of 20% sodium carbonate was added and thoroughly mixed. The reaction was carried out for 30 min at room temperature. Afterward, the absorbance value was measured using a spectrophotometer (Molecular Devices, SpectraMax^®^ ABS Plus, San Jose, CA, USA) at 760 nm. Gallic acid (1 mL) at various concentrations was used to construct a standardized curve for calculating the TPC of the samples, which was expressed as mg GAE (gallic acid equivalents)/g of DW [17].

#### 2.5.2. Total Flavonoid Content (TFC)

The determination of TFC was carried out using the colorimetric method with aluminum chloride, modified from the method reported in [18]. Quercetin was dissolved in 95% ethanol to create a standard quercetin solution. Subsequently, 50 μL of the extract or standard solution was mixed with 10 μL of 10% aluminum chloride hexahydrate, followed by 150 μL of 95% ethanol, 10 μL of 1 M potassium acetate, and 10 μL of 1 M potassium acetate, in sequence. The extract or standard solution (50 μL) and 10% aluminum chloride hexahydrate (10 μL) were added, followed by 150 μL of 95% ethanol and 10 μL of 1 M potassium acetate. Ethanol (95%) was used as a control group, and the reaction mixture was shaken and mixed homogeneously at room temperature for 40 min, protected from light. The absorbance value was measured using a spectrophotometer (Molecular Devices, SpectraMax^®^ ABS Plus, San Jose, CA, USA) at 415 nm. The total flavonoid content was expressed as mg QE (quercetin equivalents)/g of DW.

#### 2.5.3. Total Ascorbic Acid Content (TAAC)

TAAC was determined using the indophenol method, following the procedure outlined by Nielsen and Nielsen [19]. In this method, the extract was combined with a metaphosphoric acid–acetic acid solution in a 1:1 (*v*/*v*) ratio to create a sample solution. Ascorbic acid was then added to the metaphosphoric acid–acetic acid solution to serve as the standard solution. Metaphosphoric acid–acetic acid solution (7 mL) was used as a blank test. A standard group was prepared using 2 mL of ascorbic acid standard solution and 5 mL of metaphosphoric acid–acetic acid solution. The standard solution was calibrated with indophenol (2,6-dichloroindophenol). The sample solution (10 mL) was mixed with 5 mL of metaphosphoric acid–acetic acid solution to titrate with indophenol standard solution. Double-distilled water (ddH_2_O; 10 mL) was used as a blank group. The amount of titration was recorded and the total ascorbic acid content was calculated using the following formula:

$$TAAC(\frac{mg}{mL})=\frac{\left(a-b\right)\times D.F\times K}{V}$$

a: Number of mL of indophenol used in the sample juice;

b: Number of mL of indophenol consumed in the blank test;

D.F.: Sample dilution factor (juice diluted 1:1) = 2;

V: Sample volume;

K: $\frac{Weight\;of\;ascorbic\;acid\;(mg\;)\;\times \;\frac{2\;(mL)}{50\;(mL)}}{Ascorbic\;acid\;standard\;solution\;titration\;volume\;\left(mL\right)\;-\;Blank\;titration\;volume\;(mL)}$.

#### 2.5.4. Analysis of Flavanone Content by HPLC-PDA

The sample was filtered through a 0.22 μm microporous membrane. Then, 10 μL of the extract was injected into the HPLC. The peaks were identified based on the retention time of the standardized products (naringin, hesperidin, neohesperidin, and naringenin) and their UV spectrum. The peak areas of the sample extract were then substituted into the standard curve to calculate the flavanone content. A Sykam HPLC pump system (S1125 HPLC Pump System, S5200 Autosampler, Radebeul, Germany) with a photodiode array (PDA) detector was used. The main analyzing column was InertSustain C18 (250 mm × 4.6 mm, 5 μm pore size). The mobile phase A was acetonitrile and the mobile phase B was water. The gradient settings were as follows: 22% A and 78% B, held for 10 min, changing linearly to 61% A and 39% B (25 min), and then to 100% A (5 min). Then, it was returned to initial conditions after 5 min and was held for 15 min. The flow rate of the mobile phase was 1.0 mL/min, and the sample injection volume was 10 μL. The observed wavelength was 280 nm [20]. The intra-day test involved injecting 5, 50, and 100 μg/mL of standardized flavanone solution 3 times within 24 h at each concentration.

### 2.6. Measurement of Antioxidant Capacity

#### 2.6.1. ABTS (2,2-Azino-Bis-3-Ethylbenzothiazoline-6-Sulphonic Acid) Assay

The method was modified from that of Chew et al. [21], wherein 14 mM ABTS and 4.9 mM potassium persulfate were combined in a 1:1 ratio to create a masterbatch of ABTS radicals. The reaction was then left to stand in the dark for 12–16 h. For the analysis, 1.9 mL of ABTS radicals was mixed with 50 μL of the sample, resulting in an absorbance value of 0.70 ± 0.05 at 734 nm with a 95% ethanol dilution. Then, 1.9 mL of ABTS radical was added to 50 μL of the sample and left to react in the dark for 6 min. The absorbance value was measured at 734 nm using a spectrophotometer (Molecular Devices, SpectraMax^®^ ABS Plus, USA). The percentage of free radical scavenging ability (%) was calculated as follows:

$$ABTS\;racial\;scavenging\;activity\;(\%)=\left(\frac{A_{control}-A_{sample}}{A_{control}}\right)\times 100\%$$

A_control_: The absorbance value of the control group;

A_sample_: The absorbance value of the sample.

The control group was standardized using distilled water and various concentrations of Trolox solution and ABTS radical scavenging capacity (%) was determined, with the results expressed in μmole TE/g of DM.

#### 2.6.2. DPPH Assay

Citrus peel extract (22 μL) was taken and 200 μL of a freshly prepared 95% (*v*/*v*) ethanol solution of 150 μM DPPH was added to it. The mixture was then homogeneously mixed and left to stand for 30 min at room temperature, protected from light. The absorbance of DPPH at 517 nm was measured using a spectrophotometer (Molecular Devices, SpectraMax ^®^, ABS Plus, USA), and the radical scavenging ability (%) of DPPH was calculated as follows [22]:

$$DPPH\;radical\;scavenging\;activity\;\left(\%\right)=\left(\frac{A_{control}-A_{sample}}{A_{control}}\right)\times 100\%.$$

A_control_: Absorbance value of the control group; A_sample_: absorbance value of the sample.

In the control group, distilled water was used as a substitute for the extract samples, and we standardized the curve using different concentrations of Trolox solution and DPPH radical scavenging capacity. Standardized curves were created using various concentrations of Trolox solution and DPPH radical scavenging capacity. The results were expressed as μmole Trolox equivalents (TE)/g of DM.

#### 2.6.3. FRAP Assay

The FRAP assay was based on the method used by Benzie and Strain [23]. The FRAP reagent consisted of a mixture of 0.1 M sodium acetate buffer (pH 3.6), 10 mM 2,4,6-tri(2-pyridyl)-s-triazine (TPTZ), and 20 mM iron chloride (10:1:1 *v*:*v*:*v*). The extract (0.1 mL) was added to 1.9 mL of the reagent and the absorbance value was determined using a spectrophotometer (Molecular Devices, SpectraMax ^®^ ABS Plus, USA) at 593 nm. The change in absorbance at 593 nm, between the final reading selected and the 4 min reaction reading, was calculated for each sample and was related to the change in absorbance of a FeII standard solution tested in parallel. This was expressed as μmol Trolox equivalent antioxidant capacity (TE)/g of dry weight (DW).

### 2.7. Pectin Extract

In a slight modification of the method described by Peng et al. [24], 10 g of sample powder was washed twice with 63.3 mL of 95% ethanol to remove mono- and di-sugars [25] and mixed with 31.7 mL of acetone. The suspension was filtered, and the residue was dried at 40 °C to a constant weight to obtain a dried alcohol-insoluble residue (AIR). Referring to the methods of Kliemann et al. [26] and Pasandide et al. [27], combining one factor at a time [28], the experiment was designed as follows: the dry pomace was extracted with solvents in the ratios of 1:20, 1:25, and 1:30 (*w*/*v*), and the pH values were adjusted to 1, 2, and 3 with 0.5 M of HCl, H_2_SO_4_, and citric acid, respectively, while the control group was extracted aqueously with an equal volume of water instead of acid. Then, 0.5 M of HCl, H_2_SO_4_, and citric acid were used to adjust the pH values to 1, 2, and 3, respectively, while the control group was extracted in water by replacing the acid with an equal volume of water. The mixtures were extracted by heating to 70 °C, 80 °C and 90 °C for 10, 20 and 30 min. The mixtures were centrifuged in a tabletop centrifuge (Kubota, RA-2024, Bunkyo, Tokyo, Japan) and the supernatant and solids were collected separately. The filtrate was cooled down to 4 °C and allowed to stand for 1 h. An aliquot of 95% ethanol was added to the filtrate, followed by the addition of an aliquot of 70% acidic ethanol (0.5% HCl), then washed with 95% ethanol to pH 7, filtered to remove the supernatant, and dried in a hot-air oven for 2 h at 35 °C.

### 2.8. Total Dietary Fiber (TDF)

The assay was carried out according to the method outlined by the AOAC [29]. First, 1 g of the sample (S) was weighed in duplicate and added to 50 mL of pH 6.0 phosphate buffer. Then, 100 μL of Termamyl solution was added, and the mixture was placed in a boiling water bath for 15 min. After cooling, 10 mL of 0.285 N NaOH solution was added, and the pH value was adjusted to 7.5 ± 0.1. After cooling, 10 mL of 0.329 M phosphate solution was added to adjust the pH value to 4.5 ± 0.2. Then, 5 mg of protease was added and stirred at 60 °C for 30 min. After cooling, 10 mL of 0.329 M phosphoric acid was added to adjust the pH to 4.5 ± 0.2. Amyloglucosidase (0.3 mL) was added and stirred at 60 °C for 30 min. Ethanol (95%; 280 mL) was added and preheated to 60 °C, then the mixture was allowed to stand at room temperature for 60 min to form a precipitate, then the precipitate was dried and weighed (R). After weighing the fiber cups, the samples were lubricated with 74% ethanol. The enzyme-treated samples were added to the fiber cups and the residue was washed with 3 portions of 20 mL with 74% ethanol, 2 portions of 10 mL with 95% ethanol, and 2 portions of 10 mL with acetone. Subsequently, the samples were dried overnight at 70 °C. The first sample underwent Kjeldahl analysis, wherein the first sample was analyzed for Kjeldahl nitrogen (P%), and the second sample was ashed and weighed at 520 °C (A%). Calculations were made using the following formula:

$$TDF\left(\%\right)=\frac{R-\frac{\left(P\%+A\%\right)}{100\times R}-Blank}{S}\times 100(\%)$$

### 2.9. Statistical Analysis

Before analyzing the data collected from this research with a one-way ANOVA, it was confirmed that the assumptions of normality, equal variances, and independence were adequately met. The mean values of the experimental data were calculated by conducting triplicate measurements, utilizing a one-way analysis of variance and Duncan’s multiple range test to assess the degree of difference between the experimental data, assuming a significance level of *p* < 0.05. Data were analyzed using the SPSS 22.0 (Statistical Product and Service Solutions) package (SPSS Statistical Software, Inc., Chicago, IL, USA).

## 3. Results

### 3.1. Drying Results for Ponkan By-Products

The moisture content of ponkan by-products before drying was 64.38%, making it difficult to preserve the fresh samples as this led to fruit-fly infestation. Therefore, the samples were dried for storage in this study. The yield after microwave vacuum drying (MVD) was 27.53%, while after freeze-drying (FD), it was 26.13%, and after hot air drying (HAD), it was 27.91%. There was no significant difference between them. The shortest drying time for one kilogram of ponkan by-products was with MVD (78.6 min), followed by HAD (1718 min), and FD (4320 min). Additionally, there was no significant difference in the water activity of FD and HAD, while MVD was slightly higher than both. According to the study by Tapia et al. [30], a water activity level below 0.61 is considered to prevent microbial proliferation.

### 3.2. Proximate Analysis of Ponkan By-Products

The moisture content of ponkan by-products was 64.38%, similar to the dried results. The ash and crude fat contents were 4.20% and 0.62%, respectively, which aligns with the findings of Boluda-Aguilar et al. [31], wherein moisture was identified as the major component. The crude protein and crude fiber contents were 2.31% and 10.7%, respectively. The total carbohydrate content was 28.49%, which differed from the findings of Kaushal et al. [32]. This variance could be attributed to differences in geographic origin, harvesting time, and other factors.

### 3.3. Results for the Phytochemical Content of Ponkan By-Products by Extractive Solvents

**Figure 1:**
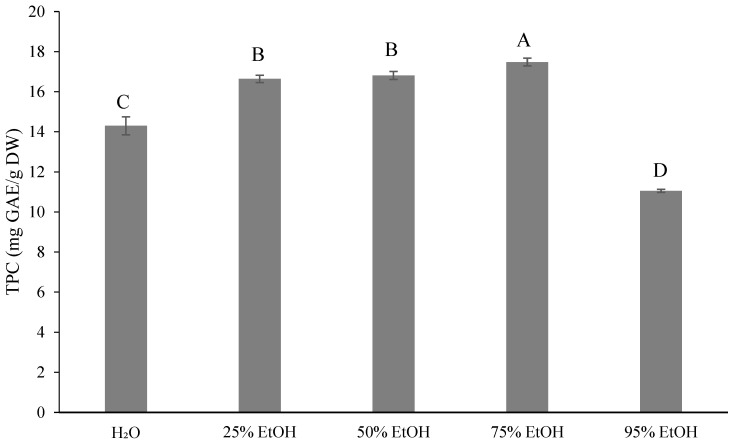
Total phenolic contents (mg GAE/g dry weight) for different ethanol volumes used for extraction from *Citrus reticulata* by-products. All data are shown as means ± SD (*n* = 3). Different letters represent significant differences (*p* < 0.05). TPC, total phenolic contents. GAE, gallic acid equivalents.

**Figure 2:**
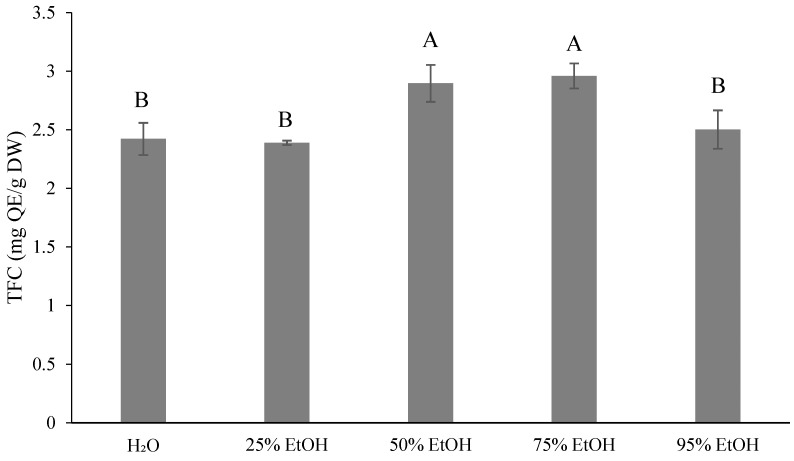
Total flavonoid contents (mg QE/g dry weight) for different ethanol volumes used for extraction from *Citrus reticulata* by-products. All data are shown as means ± SD (*n* = 3). Different letters represent significant differences (*p* < 0.05). TFC, total flavonoid contents. QE, quercetin equivalents.

**Figure 3:**
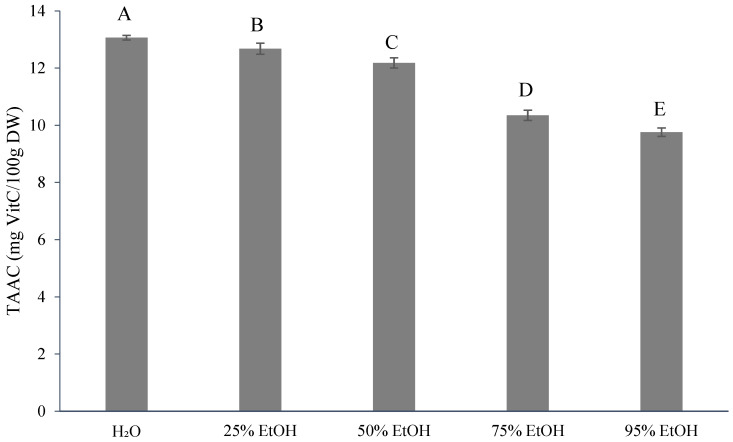
Total ascorbic acid contents (mg Vit C/100 g dry weight) for different ethanol volumes used for extraction from *Citrus reticulata* by-products. All data are shown as means ± SD (*n* = 3). Different letters represent significant differences (*p* < 0.05). TAAC, total ascorbic acid contents. VitC, vitamin C.

Figure 1 shows the TPC results (11.06–17.48 mg gallic acid equivalents (GAE)/g dry weight (DW)) after extraction with five different concentrations of ethanol. In Figure 2, the total flavonoid content (TFC) is presented. The 75% ethanol extract exhibited the highest TFC (2.96 mg quercetin equivalents (QE)/g, DW), which was not significantly different from that with 50% ethanol extract (2.90 mg QE/g, DW). In contrast, the 25% ethanol extract showed the lowest TFC (2.39 mg QE/g, DW), which was not significantly different from both the control (2.42 mg QE/g, DW) and that of the 95% ethanol extract (2.50 mg QE/g, DW). The lowest TFC of 2.39 mg QE/g of DW was obtained with the 25% ethanol extract, which was not significantly different from the control group (2.42 mg QE/g, DW) and the 95% ethanol extract (2.50 mg QE/g, DW). Figure 3 illustrates the TAAC, with the highest value observed with water extraction (13.07 mg VitC/100 g, DW) and the lowest with 95% ethanol extraction (9.76 mg VitC/100 g, DW). This difference can be attributed to the water solubility of ascorbic acid, indicating that a higher solvent content in water leads to better extraction results. According to previous studies, the TAAC of oranges was 12.78 mg VitC/100 mL [33] and that of sweet oranges was 10.13 mg VitC/100 mL [34].

### 3.4. Analysis of the Flavanone Content of Ponkan by HPLC-PDA

The naringin content ranged from 16.74 to 61.83 mg/100 g of DW, which was comparable to the findings of Shamloo et al. [35] who used methanol to extract sweet orange (39.06 mg/100 g), and was similar to the results reported for ponkan peel extracted with 80% acetone (58.138 mg/100 g) [36]. However, this was lower than the naringin content reported by Ho and Lin [37] using DMSO/methanol (587 mg/100 g), which could be attributed to the variations in extraction methods and locations. The hesperidin content ranged from 102.95 to 622.57 mg/100 g DW, which was similar to that found in the orange juice extracted by ultrasonication-assisted 90% methanol extraction (546.7 mg/100 g, DW) [38]. The results for the ultrasonically-assisted methanol extraction of *Chaetomium cepa* (5526.04 mg/100 g) reported by Sun et al. [20] were higher than those in the present study, which may be due to the difference in maturity and the fact that the method for reactive surfaces was not optimized in the present study. Neohesperidin content ranged from 4.57 to 19.55 mg/100 g DW, which was comparable to the findings for the methanol/DMSO/water extraction of sweet orange (47 mg/kg) reported by Wang et al. [39]. The naringenin content varied from 0.72 to 6.69 mg/100 g DW, which was lower than the outcome of the methanol/DMSO/water extraction of sweet orange (18.3 mg/100 g) by Wang et al. [39].

Table 1 presents the flavanone content of ponkan by-products extracted using various ethanol concentrations. The extract of hesperidin exhibited the highest content, followed by naringin, neohesperidin, and naringenin. Notably, the extract with 95% ethanol had the highest concentration of naringenin. Due to its solubility in alcohol, the 95% ethanol extract had the highest content, while the water extract had a lower content. Therefore, the 95% ethanol extraction yielded the highest flavonoid content, while the water extraction was significantly less effective. This difference can be attributed to the fact that the four compounds were soluble in alcohol, indicating that a higher alcohol concentration resulted in better extraction outcomes.

### 3.5. Antioxidant Activity of the Extracted Solvent on Ponkan By-Products

**Figure 4:**
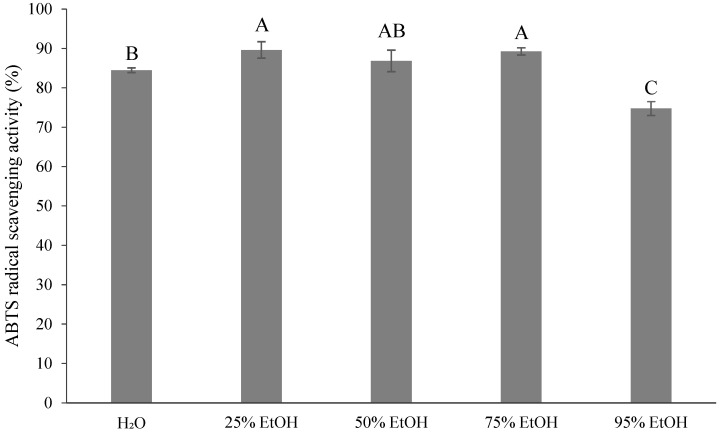
ABTS radical scavenging activity (%) for different ethanol volumes of extraction from *Citrus reticulata* by-products. All data are shown as means ± SD (*n* = 3). Different letters represent significant differences (*p* < 0.05). ABTS: 2,2′-azino-bis(3-ethylbenzthiozoline-6)-sulphonic acid method.

Figure 4 and Table 2 show that the ABTS radical scavenging capacity of the control group was 84.47%, or 122.16 μmol Trolox equivalent antioxidant capacity (TE)/g of DW. There was no significant difference between the 25%, 50%, and 75% ethanol extracts (86.84–89.63%). The lowest scavenging capacity was found for the 95% ethanol extract (74.76%), with 97.841 μmol TE/g of DW. Anticona et al. [40] reported that the ABTS radical scavenging capacity of UAE with hybrid ponkan was 18.1–30.4 mmol TE/100 g, which was higher than the results of the present study. This difference could be attributed to variations in the varieties, extraction methods, and extraction solvents used.

**Figure 5:**
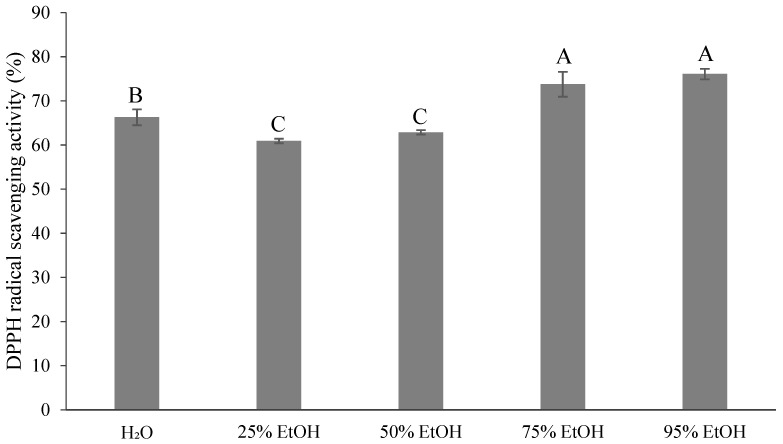
DPPH radical scavenging activity (%) for different ethanol volumes of extraction from *Citrus reticulata* by-products. All data are shown as means ± SD (*n* = 3). Different letters represent significant differences (*p* < 0.05). DPPH: 2,2-diphenyl-1-picrylhydrazyl radicals method.

Figure 5 and Table 2 demonstrate that there was no significant difference between the 75% ethanol extract and 95% ethanol extract samples, which measured 23.43 and 23.73 μmol TE/g of DW, respectively. Both extracts outperformed the control group (22.65 μmol TE/g, DW), indicating that the 75% and 95% of ethanol extract effectively enhanced the antioxidant capacity. According to Zhang et al. [41], the DPPH values of ponkan were 29.04–50.46 μmol TE/g of DW, which were similar to the results of this study. The DPPH free radical scavenging ability of ponkan, as extracted by UAE and 80% methanol, was 85.53–94.65%, while that of lime peel was 86.47–94.33% [42], exceeding the results of the current study.

### 3.6. Effect of Extraction Conditions on the Pectin Yield and Results for the Phytochemical Content of Pectin Using Extraction Solvents

Existing pectin extraction conditions require large amounts of solvents and may lead to serious adverse environmental impacts, including energy consumption and poor water utilization, while the use of hydrochloric acid may lead to corrosion of stainless steel, necessitating a reconsideration of extraction methods to optimize pectin functionality and bioactivity [43]. Pectin production and characterization are affected by the source of the raw material, ripening stage, and extraction factors including pH, temperature, solvent type, and time [44,45]; therefore, the one factor at a time approach was chosen to determine the optimal solid–liquid ratio, pH, temperature, and time for pectin extraction. It is important to analyze the phytochemical content of the extracted pectin because acid extraction or hot water extraction can lead to co-extraction phenomena [46].

#### TPC and TFC

**Figure 6:**
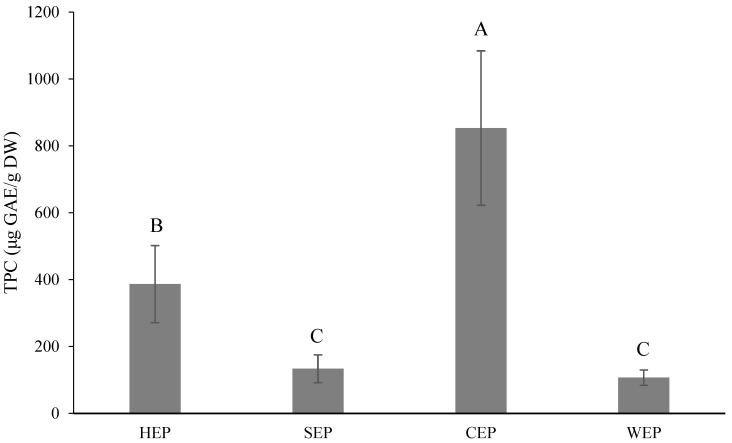
Total phenolic contents (μg GAE/g dry weight) of *Citrus reticulata* pectin with different solvents used for extraction. All data are shown as means ± SD (*n* = 3). Different letters represent significant differences (*p* < 0.05). TPC, total phenolic contents. GAE, gallic acid equivalents. HEP, hydrochloric acid extraction of pectin. SEP, sulfuric acid extraction of pectin. CEP, citric acid extraction of pectin. WEP, water extraction of pectin.

The TPC is shown in Figure 6, and the citric acid extraction of pectin (CEP) showed the highest content (853.33 μg GAE/g DW), followed by the hydrochloric acid extraction of pectin (HEP) (386.67 μg GAE/g DW), while the sulfuric acid extraction of pectin (SEP) and water extraction of pectin (WEP) showed no significant difference. Wang et al. [47] reported the TPC of grapefruit pectin extracted by an ultrasonication-assisted thermal hydrochloric acid extraction technique to be 4210 μg GAE/g, and that of thermal hydrochloric acid-extracted pectin to be 7.06 μg GAE/mg. Lin et al. [48] reported the TPC of commercial *Citrus* pectin to be 1770 μg GAE/g; all of these contents were higher than in the results of the present study, which may be caused by different varieties or extraction methods.

**Figure 7:**
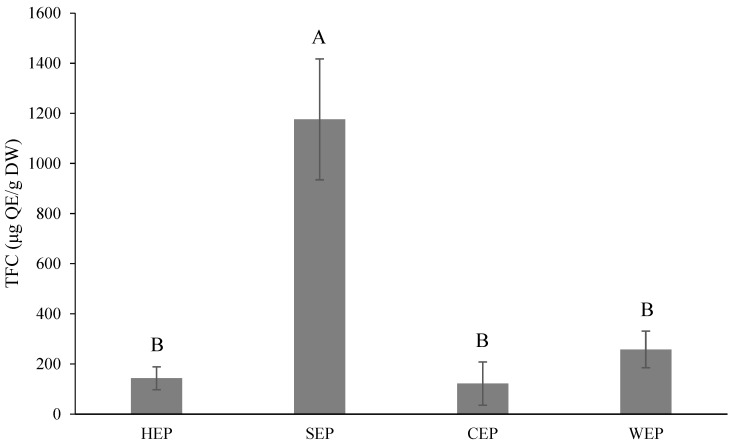
Total flavonoid contents (μg QE/g dry weight) of *Citrus reticulata* pectin with different solvents used for extraction. All data are shown as means ± SD (*n* = 3). Different letters represent significant differences (*p* < 0.05). TFC, total flavonoid contents. QE, quercetin equivalents. HEP, hydrochloric acid extraction of pectin. SEP, sulfuric acid extraction of pectin. CEP, citric acid extraction of pectin. WEP, water extraction of pectin.

The TFC is shown in Figure 7, with the highest SEP being 1176.06 μg QE/g DW, and with no significant difference among the other three (121.52–257.88 μg QE/g DW). According to Lin et al. [48], the TFC of commercial *Citrus* pectin was 1080 μg QE/g DW, which was similar to the SEP results in this study.

### 3.7. Antioxidant Activity of Pectin Extract Solvents

#### 3.7.1. DPPH

The DPPH radical scavenging ability of the pectin samples is shown in Table 3. The best DPPH radical scavenging ability was found in WEP (0.219 μmol TE/g DW), while the remaining three did not show any significant difference (0.004–0.043 μmol TE/g DW).

#### 3.7.2. FRAP

The FRAP ferric reducing antioxidant capacity of the pectin samples is presented in Table 3. The best FRAP ferric reducing antioxidant capacity was found to be for HEP and WEP, which were not significantly different from each other (13.03 μmol TE/g DW and 15.04 μmol TE/g DW), and the lowest was found to be for CEP.

### 3.8. Total Dietary Fiber (TDF) of Ponkan By-Products

The TDF content in Taiwanese Ponkan (25.70%) was similar to that reported by Chang et al. [49]. The TDF content was similar to that reported by Oduntan and Arueya [50] for sweet orange (27.69%). The residual TDF after pectin extraction was 13.39–17.02%, which was lower than that of the ponkan by-products after MVD treatment. This difference can be attributed to the extraction of soluble dietary fiber (SDF) in previous experiments. It was inferred that the remaining portion should be insoluble dietary fiber (IDF), a finding similar to that reported in the study by Oduntan and Arueya [50] (15.23%), a slightly higher figure than that reported in the study by Chang et al. [49] (10.30%). The lowest TDF content was found in the residue after citric acid extraction, while the highest was found in the residue after water extraction, which may be related to the pectin extraction content.

## 4. Discussion

### 4.1. Drying Results for Ponkan By-Products

Although freeze-drying (FD) is the optimal choice for preserving nutrients and color, the process is characterized by long drying times, high energy consumption, and high costs [51]. Conversely, hot-air drying (HAD) is the most costly in terms of drying time, leading to the degradation of flavonoids and phenolic acids, thereby diminishing the antioxidant capacity of citrus. Additionally, HAD may cause oxidation and the pyrolysis of polyphenols [52]. Since the aim of this study was to enhance the value of the by-products and preserve their antioxidant components, MVD was selected for follow-up experiments due to its lower time and cost requirements.

### 4.2. Results for the Phytochemical Content of Ponkan By-Products with Extractive Solvents

The reason for choosing ethanol over methanol as the extraction solvent is that the total phenolic content (TPC) of ethanol extraction may be higher than that of methanol extraction. This is because ethanol tends to form hydrogen bonds with the hydroxyl groups of phenolic compounds, while methanol is less prone to hydrogen bonding [53,54].

The highest TPC was obtained with 75% ethanol, which is similar to the results of 80% methanol-extracted grapefruit peel studied by Rahman et al. [55]. It is slightly lower than that reported by Zhang et al. [56] with an 80% methanol extract of ponkan peel (22.80–32.76 mg GAE/g), but lower than that reported by Ghasemi et al. [57] (172.1 mg GAE/g, DW). There was no significant difference between the 25% ethanol extract and 50% ethanol extract, while the 95% ethanol extract had the lowest content.

The TFC of ponkan rind was reported as 5.2 mg QE/g of DW [57]. In contrast, the TFC of ponkan rind extracted using ultrasound-assisted extraction (UAE) with 80% methanol was found to be 1383 mg QE/100 g [39]. These values were higher than the results obtained in this study, possibly due to variations in natural plants, different extraction conditions such as temperature, time, the solvent-to-solid ratio, the type of solvent, and the additional extraction techniques employed [58]. Londoño-Londoño et al. [13] used methanol as a solvent to extract compounds at 60 kHz, at 40 °C, and for 30 min. They also utilized methanol as a solvent, using the same frequency, temperature, and duration to extract flavonoids from *citrus* peels. The yield thus obtained was 40.25 ± 12.09 mg/g, and the total phenolic content was 19.59 ± 2.11 mg GAE/g of dry matter (DM).

The phytochemical content increased with the rising ethanol concentration. The 75% ethanol extract exhibited the highest TPC at 17.48 mg GAE/g of DW and TFC at 2.96 mg QE/g of DW. Conversely, a decreasing trend was noted for the 95% ethanol extract. Chan et al. [59] found that pure solvents were unable to ensure an average polyphenol extraction performance compared to aqueous solutions. They suggested that the low solubility in pure solvents could be due to the strong hydrogen bonding between proteins and polyphenols. However, the addition of water to the solvent increases solubility, weakening the hydrogen bond [60]. Ethanol enhances the solubility of solutes, while water speeds up their desorption from the sample [61]. Therefore, determining the optimal concentration for extraction is crucial for maximizing the phytochemical content.

Factors affecting total ascorbic acid content (TAAC) in citrus fruits include production factors, climatic conditions, fruit variety, handling procedures, and storage conditions [62]. Ascorbic acid retention is often utilized as an indicator of the overall nutritional retention of food products because it is highly susceptible to oxidation [63] and to leaching into water-soluble media during storage [64].

### 4.3. Analysis of the Flavanone Content of Ponkan by HPLC-PDA

Botanical nutrients such as flavonoids, anthocyanins, and phenolic acids have been shown to have potential health benefits in the treatment of obesity, hypertension, cardiovascular disease, and metabolic syndrome [65]. Naringin, neohesperidin, nobelitin, narirutin, and hesperidin are among the most important flavonoids isolated from citrus fruits [66], of which hesperidin and naringin are the most significant [66]. They exhibit strong antioxidant and anti-inflammatory activities, both in vitro and in vivo.

### 4.4. Antioxidant Activity of the Extracted Solvent on Ponkan By-Products

Humans are exposed to various exogenous and endogenous oxidants, such as free radicals and reactive oxygen species, from environmental and cellular metabolism. These exposures can lead to lipid peroxidation, oxidation of the polypeptide backbone, and DNA strand damage, which, in turn, can result in serious pathological conditions such as diabetes, cancer, neurodegenerative diseases, and cardiovascular diseases [67]. Oxidative damage occurs not only in the human body but also in foods containing polyunsaturated fatty acids, due to lipid peroxidation when exposed to air, light, and heat. This process leads to deterioration, discoloration, and the loss of nutritional value [68]. Nowadays, various synthetic antioxidants such as butylated hydroxyanisole (BHA) and butylated hydroxytoluene (BHT) are commonly utilized in the food industry to prevent such losses. However, their use in the food industry is questionable, due to potential health risks and toxicity [69]. Studies have also investigated health issues arising from long-term intake [70]. Therefore, it is essential to explore natural antioxidants derived from plant sources.

ABTS radical scavenging capacity is a common method used to measure the antioxidant capacity of foodstuffs in the food industry and in agricultural research [71]. The addition of potassium persulfate to ABTS resulted in a blue ABTS radical solution with a peak absorbance at 534 nm. When the ABTS radicals were reduced, the color changed from blue to colorless, indicating that the lower the absorbance, the higher the scavenging capacity of ABTS. The color changed from blue to colorless when the ABTS radicals were reduced. This indicates that the lower the absorbance value, the better the ABTS radical scavenging ability. Moreover, the ABTS radical scavenging ability is associated with the number of hydroxyl groups in the polyphenol [72].

DPPH is one of the few stable and commercially available nitrogen-containing radicals with a maximum absorbance at 517 nm. When DPPH is reduced, the color changes from violet to colorless, indicating that the lower the absorbance, the better the scavenging ability of the DPPH free radicals. The scavenging ability of the DPPH free radicals correlates with the number of hydroxyls scavenged [73]. The primary antioxidant capacity of DPPH is determined by its reaction with antioxidant components that provide hydrogen [74].

The antioxidant activity of plant extracts depends on their ability to chelate transition metal ions, especially Fe^2+^ and Cu^2+^ [75]. Metal chelating compounds and chelator complexes exhibit antioxidant activity by reducing the redox potential and stabilizing the oxidation state of metal ions [76]. Ferrozine and Fe^2+^ are chelated to form a miscible complex (ferrozine-Fe^2+^) that is red in color and exhibits maximum absorbance at 625 nm. When the extract is chelated with Fe^2+^, the content of ferrozine-Fe^2+^ decreases, resulting in a lower absorbance value. The lower the absorbance value, the stronger the antioxidant effect of ferric reduction. The DPPH radical scavenging capacity of commercially available *Citrus* pectin was 3.91% [77]. The DPPH scavenging activity of pectin is also affected by GalA content and molecular weight, with a high GalA content and low molecular weight helping to eliminate more free radicals [78].

Table 2 displays the ferric reducing antioxidant power (FRAP) of the control group at 48.93 μmol TE/g of DW. There was no significant difference observed between the 25%, 50%, and 75% ethanol extracts (ranging from 56.56 to 58.69 μmol TE/g, DW). The 95% ethanol extract exhibited the lowest value. These findings align with those of Zhang et al. [41], who investigated the activity of 14 wild ponkan species, reporting values between 26.50 and 46.98 μmol TE/g of DW. The study suggested that limonin and naringin may contribute to the enhancement of FRAP activity [79]. The FRAP ferric reducing antioxidant capacity of commercially available *Citrus* pectin was found to be 4.21 µM TE/g DW [77], which was lower than in the results of this study.

Polyphenols and flavonoids are considered impurities in pectin. Therefore, the TPC, TFC, and antioxidant capacity of pectin extracted in this study were significantly lower than that of ponkan by-products, suggesting that 75% ethanol is effective in removing impurities to extract HMP. However, the antioxidant capacity of pectin is still present, which may be due to the presence of antioxidant components other than phenolics and flavonoids. According to previous studies, GalA has good antioxidant activity [77], and the carboxyl group of galacturonic acid may act as a hydrogen donor and electron transfer agent to promote free radical scavenging potential [80].

The ABTS radical scavenging ability (~89.63%) of ponkan peels showed better efficacy than that of DPPH radical scavenging ability (~76.08%), which may be attributed to the distinct scavenging mechanisms of ABTS and DPPH radicals [71]. Barreca et al. [81] specifically mentioned that DPPH radical scavenging ability is attributed to the action of flavonoids, so a low TFC results in relatively low DPPH radical scavenging ability. Even so, the 75% ethanol extract has excellent antioxidant activity, which may be related to the retention of more phenols, flavonoids, and carotenoids [82]. However, antioxidant activity may not always correlate with phenolic content [57], as some phenols may not have good radical scavenging ability [83].

### 4.5. Total Dietary Fiber (TDF) in Ponkan By-Products

Increasing the intake of soluble dietary fiber (SDF) and insoluble dietary fiber (IDF) is effective in reducing the risk of cardiovascular disease, gastrointestinal disease, colon cancer, glycemic response, and obesity [84]. Dong et al. [85] suggested that insoluble fibers derived from fruits and vegetables may reduce the concentration of cholesterol in the blood. Therefore, utilizing dietary fibers from plants has many health benefits. The TDF content in Taiwanese ponkan (25.70%) was similar to that reported by Chang et al. [49]. The TDF content in sweet orange (27.69%) was also similar to that found by Oduntan and Arueya [50]. A previous study has suggested that the fiber in oranges can add a soft texture to ice cream and slow down the melting rate. Additionally, the sweet and sour nature of citrus and the color of the pulp affect the quality of the ice cream. Therefore, adding oranges to ice cream creates a new flavor and increases the nutrient content [86]. Dietary fiber consumption reduces the risk of intestinal and gastrointestinal diseases, obesity, diabetes, cardiovascular disease, and cancer. It also promotes physiological functions such as the lowering of blood cholesterol levels and glucose decline [87]. Studies have shown that women need an average of 21–25 g of fiber per day, while men need 30–38 g per day [88]. The residue generated from this study could be used in ice cream in the future to enhance the value of ponkan by-products.

## 5. Conclusions

Ponkan has good antioxidant activity. Its total phenolic content (TPC) and total flavonoid content (TFC) increased with a rise in ethanol concentration. Particularly, the 75% ethanol extract exhibited the highest values (17.48 mg GAE/g, DW, and 2.96 mg QE/g, DW) and demonstrated the strongest antioxidant activity. In contrast, the 95% ethanol extract was less effective than the 75% extract. The total ascorbic acid content (TAAC) increased with a rise in water content. The highest TAAC (13.07 mg VitC/100 g, DW) was obtained with the water extract, while the lowest (9.76 mg VitC/100 g DW) was obtained with the 95% ethanol extract. This difference may be attributed to the superior antioxidant properties of ponkan by-products. The highest TAAC content was achieved with the water extract (13.07 mg VitC/100 g DW), while the lowest was with the 95% ethanol extract (9.76 mg VitC/100 g DW). This variation may be attributed to the fact that ascorbic acid is a water-soluble compound. The HPLC-PDA analysis of flavanones revealed that hesperidin was the most abundant, followed by naringin, neohesperidin, and naringenin. Due to its solubility in alcohol, the 95% ethanol extract achieved the highest content, while the water extract achieved a lower content.

The use of 75% ethanol can not only extract the most active ingredients of ponkan by-products, potentially leading to the development of functional foods and natural antioxidants in the future, but it can also serve as a solvent before pectin extraction to optimize utilization and minimize waste. The residue of the extracted pectin can be utilized as a dietary supplement or added to ice cream to enhance the value of ponkan by-products. Although microwave vacuum drying was chosen for these experiments due to cost and time considerations, the active ingredients retained by freeze-drying and hot-air drying differ. Therefore, exploring these active substances and physiological activities in the future is warranted. In these experiments, only the pectin was extracted. In future experiments, we can attempt to emulsify it or incorporate it into a jelly for various applications. The residue from the extracted pectin has been documented as being used in ice cream production. This could lead to the development of high-fiber ice cream with ponkan flavor in the future.

## Figures and Tables

**Table 1:** Flavanone contents (mg/100 g, DW) for different ethanol volumes used for extraction from *Citrus reticulata* by-products.

Solvents	Naringin	Hesperidin	Neohesperidin	Naringenin
H_2_O	16.74 ± 4.88 ^e^	102.95 ± 12.54 ^e^	4.57 ± 1.83 ^d^	0.72 ± 0.16 ^c^
25% EtOH	35.98 ± 1.83 ^d^	329.67 ± 21.23 ^d^	6.89 ± 2.31 ^c^	1.14 ± 0.71 ^c^
50% EtOH	43.20 ± 2.36 ^c^	377.64 ± 14.29 ^c^	7.37 ± 1.96 ^c^	2.89 ± 0.59 ^b^
75% EtOH	56.17 ± 3.32 ^b^	496.73 ± 9.83 ^b^	12.61 ± 2.21 ^b^	2.76 ± 0.32 ^b^
95% EtOH	61.83 ± 0.79 ^a^	622.57 ± 34.50 ^a^	19.55 ± 3.85 ^a^	6.69 ± 1.08 ^a^

All data are shown as means ± SD (*n* = 3). Different letters represent significant differences (*p* < 0.05).

**Table 2:** Antioxidant activities for different ethanol volumes of extract from *Citrus reticulata* peels, as evaluated by the ABTS, DPPH, and FRAP methods.

Solvents/Treatment	ABTS (μmol TE/g DW)	DPPH (μmol TE/g DW)	FRAP (μmol TE/g DW)
H_2_O	122.16 ± 1.48 ^b^	22.65 ± 0.27 ^b^	48.93 ± 4.24 ^b^
25% EtOH	135.09 ± 5.24 ^a^	21.76 ± 0.08 ^c^	56.56 ± 2.19 ^a^
50% EtOH	128.09 ± 6.84 ^ab^	22.36 ± 0.07 ^b^	58.47 ± 3.25 ^a^
75% EtOH	134.14 ± 2.29 ^a^	23.43 ± 0.46 ^a^	58.69 ± 3.84 ^a^
95% EtOH	97.84 ± 4.45 ^c^	23.73 ± 0.20 ^a^	26.54 ± 2.23 ^c^

All data are shown as means ± SD (*n* = 3). Different letters represent significant differences (*p* < 0.05). ABTS radical scavenging activity, DPPH radical scavenging activity, and ferric reducing antioxidant power were expressed as μmol Trolox equivalent antioxidant capacity (TE)/g of dry weight (DW). ABTS: 2,2′-azino-bis(3-ethylbenzthiozoline-6)-sulphonic acid method. DPPH: 2,2-diphenyl-1-picrylhydrazyl radicals method. FRAP: ferric reducing antioxidant power.

**Table 3:** The antioxidant activities of *Citrus reticulata* pectin with different solvents used for extraction, as evaluated by the DPPH and FRAP methods.

Extraction Solvents/Treatment	DPPH (μmol TE/g DW)	FRAP (μmol TE/g DW)
HEP	0.039 ± 0.029 ^b^	13.03 ± 1.61 ^a^
SEP	0.004 ± 0.005 ^b^	9.79 ± 1.59 ^b^
CEP	0.043 ± 0.037 ^b^	6.44 ± 0.15 ^c^
WEP	0.219 ± 0.022 ^a^	15.04 ± 2.31 ^a^

All data are shown as means ± SD (*n* = 3). Different letters represent significant differences (*p* < 0.05). DPPH radical scavenging activity and ferric reducing antioxidant power were expressed as μmol Trolox equivalent antioxidant capacity (TE)/g dry weight (DW). DPPH: 2,2-diphenyl-1-picrylhydrazyl radicals method. FRAP: ferric reducing antioxidant power. HEP, hydrochloric acid extraction of pectin. SEP, sulfuric acid extraction of pectin. CEP, citric acid extraction of pectin. WEP, water extraction of pectin.

## Data Availability

The original contributions presented in the study are included in the article, further inquiries can be directed to the corresponding author.
